# Exploring Human‐Wildlife Coexistence Through the Lens of Value Pluralism Among Indigenous Peoples and Local Communities (IPLCs)

**DOI:** 10.1002/ece3.74507

**Published:** 2026-10-14

**Authors:** Anuradhi Dulangi Jayasinghe, Georgette Leah Burns, Duan Biggs

**Affiliations:** ^1^ School of Environment and Science, Griffith Institute for Human and Ecosystem Resilience Griffith University Nathan Queensland Australia; ^2^ Australian Research Centre for Human Evolution Griffith University Nathan Queensland Australia; ^3^ Resilient Conservation, Rob Walton School of Conservation Futures Arizona State University Tempe Arizona USA; ^4^ Centre for Adaptable Western Landscapes & School of Earth and Sustainability Northern Arizona University Flagstaff Arizona USA; ^5^ Centre for Sustainability Transitions Stellenbosch University Stellenbosch South Africa

**Keywords:** conflict, conservation, governance, human–wildlife coexistence, indigenous peoples, local communities

## Abstract

A nuanced understanding of human–wildlife coexistence (HW‐coexistence) is necessary to develop effective conservation approaches that are socially just and culturally sensitive. Indigenous Peoples and Local Communities (IPLCs) are central to such efforts, given their diverse perspectives and values regarding interacting with wildlife. This paper focuses on understanding how pluralistic values of IPLCs can inform more nuanced perspectives of human–wildlife coexistence. Through a critical interpretive synthesis (CIS), we showed how the value pluralism of IPLCs begets and enriches the complexities and dynamics of coexistence. We identified a range of values held by IPLCs, such as reciprocity, tolerance, trust, and acceptance, among others. Finally, we developed a conceptual framework grounded in value pluralism that portrays the fluid and overlapping positive, negative, and ambivalent relationships between IPLCs and wildlife. The framework can act as a conceptual tool in conservation decision‐making to construct and implement inclusive and adaptable conservation strategies that acknowledge the inherent value pluralism within IPLCs interacting with wildlife.

## Graphical abstract

This paper examines how the value pluralism of Indigenous Peoples and Local Communities shapes their relationships with wildlife and governing institutions. Through a critical interpretive synthesis (CIS), we showed how the value pluralism of IPLCs begets and enriches the complexities and dynamics of coexistence. Acknowledging value pluralism, the different relationships arising from it, and the associated dynamics and complexities of coexistence must be respected and accommodated to ensure effective governance of human–wildlife interactions and achieve coexistence benefits.

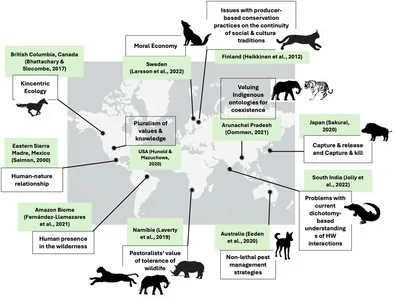

## 1 Introduction

Coexistence is one of the emerging orientations of human–wildlife interactions (Peterson et al. 2013; Pooley 2021) and refers to a dynamic yet sustainable state in which humans and wildlife adapt to live together in shared landscapes (Mekonen 2020). This is the ability of humans and wildlife to change their behaviour, learn from experience, and pursue their interests while respecting each other (Carter and Linnel 2016; Mekonen 2020). There are potential benefits to achieving conservation targets by moving beyond traditional conservation approaches (e.g., strictly separating humans and wildlife for species protection and population recovery) and shifting attention towards coexistence when conserving endangered species (Peterson et al. 2010; König et al. 2020). Dawson et al. (2021) suggest that the primary pathway to effective and long‐term biodiversity conservation is through focusing on the governance type and quality and fostering the solutions that reinforce the role of Indigenous Peoples and Local communities (IPLCs). The ontologies, knowledge systems and worldviews of IPLCs are rooted in strong interconnections between their culture and nature, which are sustained through social norms, livelihoods, language, and beliefs (Dhee et al. 2019; Toncheva and Fletcher 2022). According to Jolly and Stronza (2025, 2), ‘coexistence thrives in communities and places where relationships and connections are nurtured, and human exceptionalism is challenged’. Hence, understanding the nuances of these relationships of IPLCs and wildlife can pave novel pathways to better understand coexistence and thereby towards effective conservation (Green et al. 2024; Jolly and Stronza 2025).

Human interactions with wildlife are complex and contextual and need to be governed by effective institutions (e.g., community‐based management arrangements) to ensure social legitimacy, tolerable levels of risks, and sustainability of wildlife populations (Mekonen 2020; Woolaston et al. 2021). Castillo‐Huitron et al. (2020), suggest that many factors influence human–wildlife relationships. Notably, these include, uses, values, and meanings of animals that people perceive through time and space across cultures. Furthermore, emotional reactions towards wildlife have been identified as derived from the cultural behaviours of the societies (Castillo‐Huitrón et al. 2020). As studies on Human Wildlife‐coexistence (HW‐coexistence) continue to increase (Venumière‐Lefebvre et al. 2022), understanding the nuances among different stakeholders remains indispensable for decision‐making (Glikman et al. 2021; Venumière‐Lefebvre et al. 2022). Further, a nuanced understanding of the diverse values, perspectives, and relationships that stakeholders hold with wildlife and how these influence coexistence is pivotal. For instance, elephant management in South Africa reflects nuances among different stakeholders. While elephant culling is permitted under South Africa's National Norms and Standards as a last‐resort population management tool when elephants harm tourism experiences and pose a threat to humans, conservation groups oppose culling because African savanna elephants ( *Loxodonta africana* ) are globally endangered (Slotow et al. 2021).

The term IPLC used in this study is based on the definition by The Intergovernmental Science‐Policy Platform on Biodiversity and Ecosystem Services (IPBES): ‘individuals and communities who are, on the one hand, self‐identified as Indigenous and, on the other hand, are members of local communities that maintain inter‐generational connection to place and nature through livelihood, cultural identity and worldviews, institutions and ecological knowledge’ (IPBES 2020, para.6). Historically, the fortress conservation model (which seeks to protect biodiversity by strictly excluding people from nature) has been associated with violence against Indigenous communities and continuously displaced and destroyed their cultures (Luthy 2023). Luoma (2023) termed these injustices on IPLCs through the creation of protected areas as conservation violence. The importance of focusing on IPLCs is multifaceted. For instance, focusing on IPLCs leads to more equitable knowledge production by incorporating diverse world views and experiences, while also supporting effective conservation and biodiversity outcomes (Dawson et al. 2021). Also, IPLCs have been recognised in many cases as both the direct (when they bear immediate costs or benefits of living alongside wildlife) and indirect stakeholders (when they are affected by broader conservation governance arrangements and policies) of human–wildlife conflict and conservation governance (Dudley et al. 2022).

The plurality of values and moral worldviews that influence IPLCs' interactions with wildlife remains inadequately explored in the coexistence literature. Diverse stakeholders influence how human–wildlife interactions (HWI) are understood and managed, thus creating challenges for coexistence (Lute and Carter 2020). Similarly, Glikman et al. (2021) emphasise the importance of exploring the diverse values towards wildlife to understand the acceptance of wildlife conservation. Also, understanding value pluralism both within and among different stakeholders is essential for understanding the complexity of HWI and informing the institutions that govern them. This is because human interactions with wildlife are complex and contextual (Mekonen 2020; Woolaston et al. 2021). For instance, elephant management in South African demonstrate how stakeholder values and perceptions can differ significantly. While some stakeholders view elephants as a conservation priority, others perceive them as a threat to human safety or as a valuable tourism asset (Slotow et al. 2021). These differing perspectives illustrate the contextual nature of HWI and contribute to the complexity of wildlife management.

In this paper, based on Mekonen's (2020) definition of coexistence as humans and wildlife co‐adapt to living in shared places while being governed by effective institutions, we aimed to understand how value pluralism of IPLCs begets different relationships and influences coexistence. Effective institutions, defined here, are the formal and informal governance arrangements across local, regional and national scales that facilitate inclusive decision‐making for conservation while recognising and acknowledging diverse knowledge systems and values of those being governed. Through a literature review followed by a critical interpretive synthesis, we developed a framework with fluid borders to reflect how pluralistic values of IPLCs are enacted through or shaped by different types of relationships with wildlife and governing institutions.

## 2 Methodological Approach

### 2.1 Critical Interpretive Synthesis

We used a critical interpretive synthesis (CIS) method, which is a form of literature review that enables an understanding of the value pluralism of IPLCs to coexist with wildlife while iteratively browsing, screening, and analysing the existing literature. The CIS approach enables the construction of new conceptual understandings by transforming existing evidence and concepts while concurrently and iteratively reviewing the literature (Dixon‐Woods et al. 2006; Moat et al. 2013). This approach allows the literature to become an object of scrutiny at diverse levels of concepts (Dixon‐Woods et al. 2006; Hoelting 2022) and, importantly, allows translating studies into one another by comparing concepts/metaphors in one study with those in others (Flemming 2009).

Commencing with precise review questions was not desirable in this study, as the aim was to contribute a more nuanced and in‐depth understanding of the coexistence literature. In contrast to the conventional systematic review methodology, where specific boundaries are defined to specify inclusion criteria for papers, interpretive reviews use more diffuse boundaries that sometimes overlap with other fields (Dixon‐Woods et al. 2006). Further, we employed a purposive sampling guided by theoretical relevance where the sources were based on their relevance to conceptual development around value pluralism and coexistence, since the aim of the interpretive synthesis is to develop new conceptual insights and theory rather than summarising data. Hence, CIS allowed for a reflexive and critical engagement with diverse sources of literature, including grey literature, which is often absent in rigid systematic approaches. Such rigid approaches are ill‐suited to achieve the aim of this study, which is value‐laden and with contesting definitions and literature spanning from multiple sources to inform about marginalised voices such as IPLCs. Depending on the context, how communities perceive interactions between humans and wildlife could be different (Frank and Glikman 2019). For instance, depending on the wildlife species involved, location of the study taking place (protected vs. non‐protected land), the interpretation of human wildlife conflict and coexistence differs (Peterson et al. 2010). In this CIS process, although the full transparency of the review was not possible (Dixon‐Woods et al. 2006), the rigour of this methodology was maintained through iterative reading and reflexivity and positionality, which foreground the author's interpretive role to understand coexistence in IPLC values when interacting with wildlife. To mitigate bias, we deliberately included underrepresented sources (e.g., grey literature) and diverse perspectives in the purposive sampling.

Two academic databases (Google Scholar and Scopus) were searched from March to June 2023 for sources published between 2010 and 2023. Google Scholar was included as it captures a broader range of interdisciplinary scholarship and reports relevant to HW‐coexistence, which are often not comprehensively indexed in other databases such as Scopus. Although the dynamic search algorithms of the Google Scholar do not allow its results to be fully replicable, its use was consistent with the study's purpose and the iterative nature of the CIS. In addition to the two academic databases, non‐academic journal articles and online news‐media articles were also screened (purposive sampling for the same search terms) (Figure 1). Combinations of five search terms were used: ‘human–wildlife coexistence’, ‘human–wildlife coexistence strategies’, ‘human–nature relationships’, ‘human–wildlife interactions and conservation’ and ‘Diverse values of IPLCs’. For the initial identification, we purposely prioritised the literature appearing within the first five pages of search results. A summary of the articles reviewed during the CIS is presented in Table 1.

**FIGURE 1:**
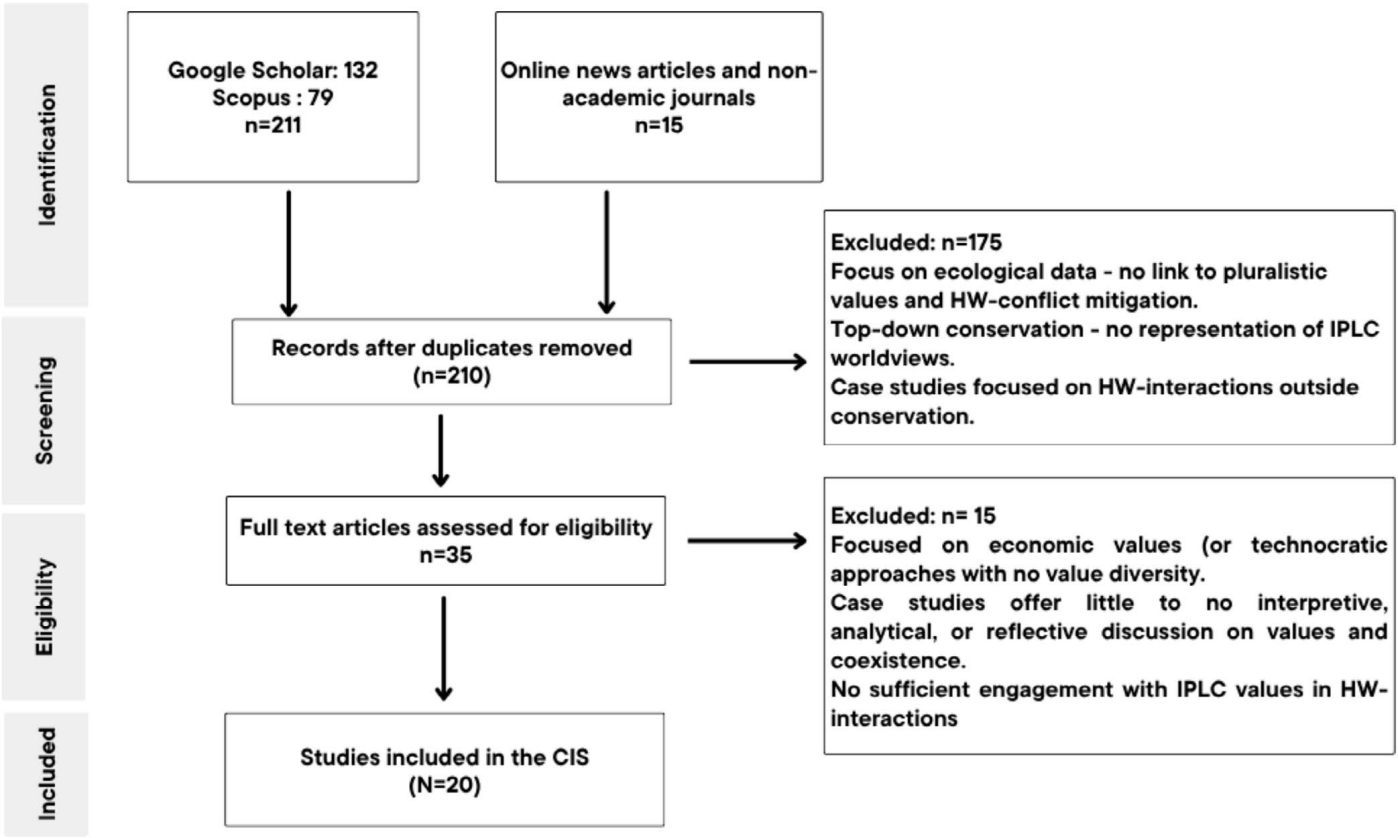
Flow chart of the literature selection process. *Source:* authors.

**TABLE 1:** A summary of the peer‐reviewed articles used for the Critical Interpretive Synthesis (CIS).

Author(s) and year	Title	Geographic region/s	Key outcomes/concerns	Stakeholder involvement	Method
Fletcher et al. (2021)	Indigenous knowledge and the shackles of wilderness	Indigenous cultural landscapes of the Amazon	The importance of recognising Indigenous and local peoples' values in dominant global conservation policy by arguing the need for human presence in the wilderness	Indigenous and local people of the selected landscapes	Case studies
		Tropical Asia and New Guinea			Wilderness and biodiversity hotspot mapping
		Australian arid lands			
Braczkowski et al. (2023)	The unequal burden of human–wildlife conflict	Global	The disparities associated with the vulnerability to conflict arising from large carnivores on cattle ( *Bos taurus* ) globally	Cattle keepers in developing economies	The proportion of Area of Habitat (AOH) of 18 large carnivore species within low, intermediate, high, and severe vulnerability related to a calf depredation event.
					Estimating economic disparities, food security impacts
Oommen (2021)	Beasts in the garden: human–wildlife coexistence in India's past and present	India	The study argues how environmental history, political ecology and anthropology can improve the understanding of human–wildlife coexistence	Locals and Mishmi communities in Arunachal Pradesh	A review using an interpretive approach, numerous empirical sources ranging from historical and anthropological accounts to recent work from conservation science that addresses the issue of coexistence.
Hoelting (2022)	Towards value pluralism, knowledge pluralism and recognitional justice: improving integration of cultural benefits of ecosystem services in environmental decision‐making	USA and global in general	Value and knowledge pluralism from sustainable conservation and coexistence	Ecosystem service beneficiaries, including Indigenous community members (e.g., Lower Elwha Klallam Tribe—LEKT)	Critical interpretive synthesis
					Interviews
Eeden et al. (2020)	A theory of change for promoting coexistence between dingoes and livestock production, conservation science and practice	Australia	A combination of appropriate monitoring and evaluation, raising public awareness, more inclusive decision‐making groups, and messaging tailored to suit rural social norms and cultures may form important steps to shifting towards evidence‐based, nonlethal policy and management.	Australian public Aboriginal people Policy makers Livestock sector	Theory of Change (ToC) to promote coexistence between livestock producers and dingoes in Australia
Laverty et al. (2019)	Using pastoral ideology to understand human–wildlife coexistence in arid agricultural landscapes, Conservation Science and Practice	Kunene Region of the Namib Desert in Nambia	How values and ecological conditions shaped individuals': (a) interactions with and tolerance of species; and (b) perceptions of challenges and potential solutions to living with wildlife	Pastoralists	Face‐to‐face semi‐structured interviews
Pooley et al. (2020)	Rethinking the study of human–wildlife‐coexistence	India and Africa	Why current human–wildlife focused on conflict, hinder the study of coexistence	Villages from study areas who encounter wildlife attacks (crocodiles—India; leopards—Africa)	Case studies
Heikkinen et al. (2012)	Users or producers of ecosystem services? A scenario exercise for integrating conservation and reindeer herding in northeast Finland	Finland	Emerging novel solutions to conservation‐pastoralism problems by seeing pastoral communities as potential producers of ecosystem services.	Pastoral communities	Ecosystem services approach: Findings from three research projects in a scenario exercise.
Bhattacharyya and Slocombe (2017)	Animal agency: wildlife management from a kincentric perspective	British Columbia, Canada	Animal agency and kin‐centric ecology are part of Indigenous cultural perspectives, worldviews, and ways of knowing in many old societies, and they have powerful implications for how humans relate to and use wildlife, lands, and resources. Yet discourse on how those Indigenous perspectives and ways of knowing might be included in, or inform alternatives to, contemporary management practice is new and still considered novel among many practitioners.	The Xeni Gwet'in First Nation	Qualitative, ethnographic methods of participatory observation, semi‐structured key informant interviews, documented discussions, and observations of horses and other wildlife.
Larsson et al. (2022)	Contextualising negative attitudes to wildlife and wildlife governance in the moral economy of Swedish farmers	Sweden	The moral economy of farming because in addition to being an economic activity, farming is integrated with values, heritage, and relations to other human beings and animals	Swedish farmers	In‐depth interviews, telephone questionnaires
Bennett et al. (2022)	Addressing the Swedish large carnivore controversy: identifying roadblocks in collaborative governance to reduce conflict	Sweden	Issues related to the governance structure and relationships within management are due to a lack of participation and trust. These must be addressed to create a socially viable collaborative governance regime to manage conflicts.	Stakeholders at different levels of wildlife management	The abductive approach combines empirical data from interviews and theories of HWC and governance.
Woolaston et al. (2021)	A review of the role of law and policy in human–wildlife conflict	Global	The study revealed a reveal a knowledge deficit on the detailed effects of law on HWC and conservation conflicts in general	Laws, national level legislations, and tribal law from identified relevant human–wildlife conflict studies	Systematic quantitative review
Hunold and Mazuchowski (2020)	Human–wildlife coexistence in urban wildlife management: insights from nonlethal predator management and Rodenticide Bans	USA	The analysis of US urban wildlife management plans and rodent control strategies reveals a shift from conflict to coexistence as the basis for understanding human–wildlife relations in urban settings.	US urban wildlife management plans and rodent control strategies	Interpretive policy analysis
Rasmus et al. (2020)	Mission impossible? Pursuing the co‐existence of viable predator populations and sustainable reindeer husbandry in Finland	Finland (reindeer management area)	Pursuing co‐existence in the present situation will require the development of novel coping strategies by herders to prevent damage and the adaptive management of predator populations.	Reindeer herders Finnish Predator Management Policy	Qualitative content analysis
Taylor‐Brown et al. (2019)	The impact of human activities on Australian wildlife	Queensland Australia	Determined the major factors driving admissions and morbidity of native animals in a region experiencing rapid and prolonged urban expansion	Wildlife rehabilitation centres	An analysis of admissions and outcomes data from a WRC in Queensland, Australia Zoo Wildlife Hospital
Manfredo et al. (2016)	Implications of human value shift and persistence for biodiversity conservation	USA (selected 19 states)	A study of value persistence and change and the nature of attitudinal responses regarding wildlife conservation issues.	State residents	Survey sampling (Schwartz value survey, mail survey)
Jolly et al. (2022)	Indigenous insights on human–wildlife coexistence in Southern India	Wayanad district of Kerala state in India	Tolerance and acceptance of wild animals by the communities are characterised as forms of deep coexistence that involve three central ideas: wild animals as rational conversing beings; wild animals as gods, teachers, and equals; and wild animals as relatives with shared origins practising dharma.	Adivasi (indigenous people of India) communities	Interviews and transect walks
Salmon (2000)	Kincentric ecology: indigenous perceptions of the human–nature relationship	Eastern Sierra Madres of Chihuahua, Mexico	Understanding the human–nature relationship through kincentric ecology	Raramuri indigenous community	Case study
Thekaekara (2019)	living with elephants, living with people: understanding the complexities of human–elephant interactions in the Nilgiris, South India.	Nilgiris, South India	Understanding the human–wild elephant interaction to inform better decisions in developing human–wildlife shared spaces beyond protected areas	Locals who are encountering wild elephant interactions	Multi‐species ethnography approach through formal and informal interviews
Nijahawa and Mihu (2020)	Relations of blood: hunting taboos and wildlife conservation in the Idu Mishmi of Northeast India	Northeast India	Taboos play a vital role in everyday Idu life by ensuring that people understand the inextricable link between human well‐being and restrictive hunting.	Idu Mishmi community	Ethnographic fieldwork
Fernandez‐Llamazares et al. (2020)	Reframing the wilderness concept can bolster collaborative conservation	Amazon biome	Suggesting securing indigenous rights as a buffer against deforestation	Indigenous and rural communities	A review
Dayer et al. (2017)	Blaming threatened species: media portrayal of human–wildlife conflict	USA	The overall tone of the articles was mostly positive	Media coverage of the piping plover bird and its conservation	A review of media articles on framing the sources of conflicts.
Stafford et al. (2018)	Media reporting of conflict between wildlife and people spending time in nature	USA	Wildlife was blamed for conflicts, and safety tips were not included in news media articles.	Conservation organisations. Humans who are recreating in nature.	Review of news media on human–wildlife conflicts
Sakurai (2019)	A collaborative approach for coexistence with wildlife in rural regions of Japan	Japan	Local residents collaborate to cook and eat boar meat (captured around the region) to increase awareness about wildlife	Rural locals (agriculture community), urban university students	A collaborative approach to raising awareness among the two stakeholders
Sakurai (2020)	Human dimensions of Asiatic black bear conflicts and management in Japan	Japan	What types of actions were suggested by the locals—capture and kill? Capture and release?	Rural local agricultural community	Interviews, group discussions

*Source:* authors.

### 2.2 Analytical Process, Framework Development and Researcher Positionality

The first author conducted an exploratory reading of the 20 articles identified for the CIS, in search of recurring concepts related to HW‐coexistence, examples of IPLC values and their relationships with wildlife, and IPLC interactions with governing institutions. During this review of 20 full‐text articles, their in‐text citations were also examined to support the interpretations, show linkages between the examples, and ensure the flow of the paper.

The initial emergent concepts included kinship, reciprocity, trust, coexistence, and conflict management. CIS does not require formal qualitative coding; however, the emergent concepts and recurring themes were recorded in a Word document and grouped according to values, types of relationships, interactions with governing institutions and coexistence outcomes. Through iterative screening and literature review, concepts identified in one study were compared with concepts emerging from other studies. Although the first author led the literature review and synthesis, these emergent concepts and thematic groupings were regularly discussed with the second author to refine conceptual boundaries. This process revealed common patterns of describing IPLCs' relationships with wildlife, as well as with governing institutions. This led to the development of synthetic constructs representing different types of relationships, themes, and recurring concepts to reflect the aim of the study. A conceptual framework was then developed by linking these synthetic constructs to value pluralism to illustrate how different relationships influence coexistence outcomes.

The critical interpretations in this study occur in several sections. In the results section, CIS is used to show how value pluralism begets different relationships, which then lead to coexistence outcomes. Finally, the entire discussion section is built from a critical interpretation of the results. Moreover, none of the authors is a representative of the IPLCs discussed in the reviewed literature. Therefore, neither the analysis nor the framework developed claims to represent IPLCs' voices directly but rather seeks to critically interpret and synthesise published accounts while recognising IPLCs' values, knowledge and their dedication to respect and protect wildlife.

## 3 Conceptual Background

### 3.1 Value Pluralism

The concept of pluralism is defined by the coexisting diversity of fundamental perspectives, such as worldviews, values and politics, that are neither subordinate to one another nor to a single unified standard (Cortés‐Capano et al. 2022). Pluralistic approaches in addressing complex challenges, such as biodiversity conservation, are increasingly gaining attention to better capture and describe the diversity of values assigned to biodiversity (e.g., instrumental—nature as the provider; relational—emergent virtues from human–nature relations) by different stakeholders (Zafra‐Calvo et al. 2020). Consequently, monetary valuation of environmental goods and services, which has been dominant in environmental management, does not capture important aspects of human–nature relations (Isacs et al. 2022).

The concept of values is understood differently across disciplines (Rawluk et al. 2019) and values can be conceptualised in various ways, for instance, individual, shared, held, assigned, economic, or environmental (Cortés‐Capano et al. 2022). Broadly, values reflect what people individuals and communities consider desirable and often guide behaviours and attitudes (Cortés‐Capano et al. 2022; Isacs et al. 2022). The concept of value pluralism recognises the existence of multiple and often conflicting values, for instance, autonomy, knowledge, justice, equality, and beauty, but without being reduced to a single ultimate value or set in fixed ranking (Cortés‐Capano et al. 2022). This pluralism is embedded within broader value systems shaped by social norms, religious beliefs, motivations, and attitudes (Afker 2015). In the context of wildlife, value pluralism influences both individual and collective actions towards how people perceive, interact with, and relate to wildlife values (Teel et al. 2010; Arias‐Arévalo et al. 2018; Vatn et al. 2024).

### 3.2 HW‐Coexistence and Relationship Types

The interactions and relationships of humans with other humans and wildlife are ‘dynamic, adapting and changing’ (Hill 2021, 2). However, these relationships are often discussed under the dichotomy of being either positive or negative (Lozano et al. 2019; Haight et al. 2023; Ellis and Stanton 2024). The conflict and coexistence dichotomy model (Hill 2021) does not capture the nuances, complexities and dynamism of relationships between humans and humans or between humans and wildlife. Green et al. (2024) highlight that failure to incorporate these nuances in mainstream conservation practices poses an array of ethical and practical consequences. For instance, in Sagalla, Kenya, where community participation in elephant management is limited, local communities perceive that conservationists prioritise elephant needs over local communities. In this situation where elephant management lacks cultural sensitivity and inclusivity, human–elephant coexistence was perceived as coercion, thus leading to resistance and retaliatory killing of elephants (Kamau and Sluyter 2017; Green et al. 2024).

Frank's (2016) conflict‐to‐coexistence continuum model spans from extremely negative attitudes towards wildlife to positive attitudes with full integration and respect for wildlife. Although this model suggests conflicts and coexistence relating to each other with no fixed points along the continuum, it is questionable how it accommodates the complexity and dynamic nature of human–wildlife relationships. For instance, representing the model risks oversimplifying the diverse and often contradictory ways in which people relate to wildlife. People may simultaneously have both positive and negative perceptions of the same species, by valuing their cultural and spiritual significance while also experiencing fear and economic losses. Such situations cannot always be positioned on a conflict‐to‐coexistence continuum. As such, there should be room for ambivalent relationships derived from value pluralism. According to Bhatia et al. (2019), recognising ambivalence in human attitudes towards wildlife facilitates the incorporation of diversity into conservation narratives and provides a more nuanced understanding of HWI. However, Bhatia et al.'s (2019) explanation of ambivalence was based on a dichotomic view into HWI—such as conflicts are negative and coexistence is positive. Hence, they defined ambivalent responses to wildlife as where ‘individuals tend not to act either way or are undecided about how they feel towards the wild animal and its impact’ (p. 624). However, in our study, we go beyond framing IPLCs' indecisiveness towards interacting with wildlife as merely ambivalent. Therefore, we view HWI that are complex, contradictory and uncertain as ambivalent because they cannot be easily classified as positive or negative relationships. However, the ambivalent relationships discussed in this paper will encompass both positive and negative aspects and will not be treated solely as a midpoint between them.

Based on the theory of rationality (Liboiron and Lepawsky 2022), the authors understood the concepts of positive and negative relationships and interpreted ambivalent relationships as they emerged inductively through the CIS. We understood the term ambivalence in different forms. For instance, ‘ambivalence’ refers to contradictory interactions and attitudes of humans towards wildlife (Harvey and Mazzotti 2019) and neutrality and indifference in human–wildlife interactions (Lehnen et al. 2021). The surface patterns of these relationships extend beyond IPLCs and wildlife, but also between IPLCs and governing institutions when interacting with wildlife.

## 4 Results

Figure 2 shows selected examples that are geographically and species‐specific. Through the CIS, we compiled value pluralism expressed by IPLCs when interacting with wildlife and also with the institutions that govern human–wildlife interactions, which were then thematically structured into three types of relationships: positive, negative, and ambivalent.

**FIGURE 2:**
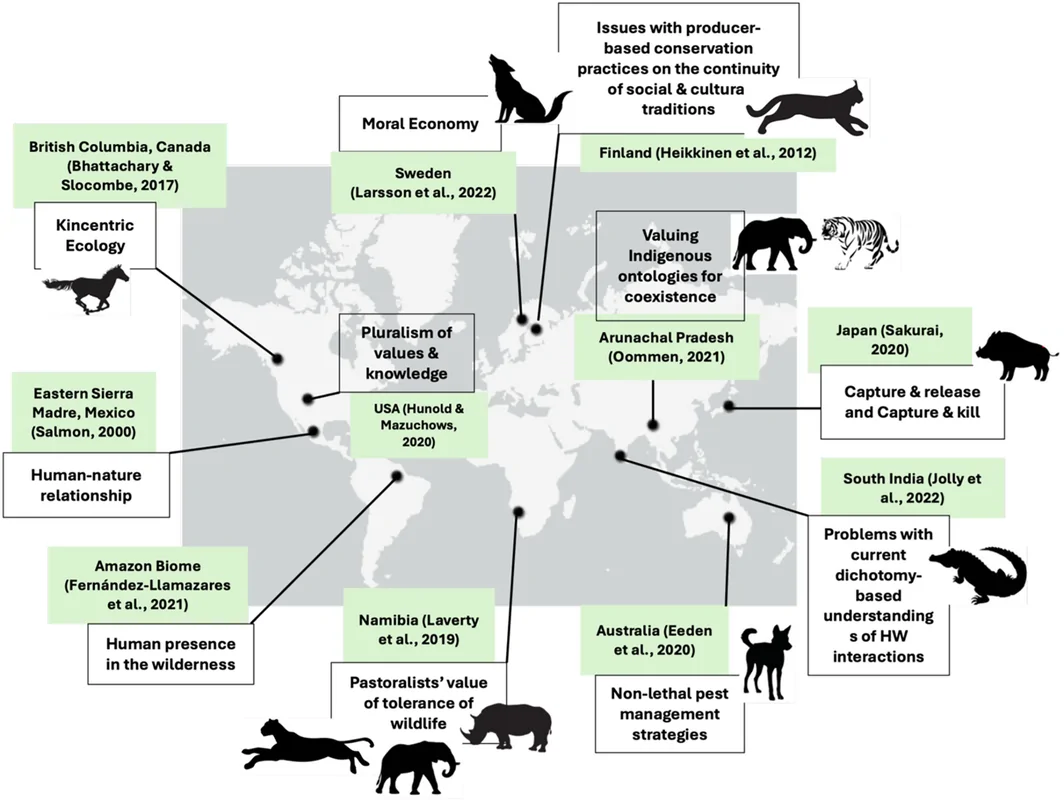
Illustrative examples of value pluralism and relationship types between IPLCs and wildlife, identified through the CIS. These examples were chosen from the 20 studies included in the CIS, as they provide clear, location‐specific and/or species‐specific empirical illustration of diverse values, relationship types, management approaches, and geographic contexts. The green boxes indicate the study location and reference source. The white boxes indicate values, conceptual insight, management approach, or the type of relationship identified in the study. The animal silhouette is the focal wildlife species discussed in the case study, while the map markers and connecting lines indicate the study's geographic location and its link to the corresponding example. *Source:* authors.

### 4.1 Positive Relationships Emerging Through Value Pluralism Towards Wildlife

#### 4.1.1 Deep Coexistence

Some cultures view animals as non‐human persons and kin (Salmon 2000; Bhattacharyya and Slocombe 2017), portraying conscious efforts of IPLCs that are beyond adaptation and co‐adaptation (Jolly and Stronza 2025), which we here define as deep coexistence. Salmon (2000, 2022) and Camou‐Guerrero et al. (2022) describe the theory of kincentric ecology through an example of the Raramuri community (also known as Tarahumara) of Mexico. In a kincentric community, people, in this case, the Raramuri community, have a belief that all life shares the same breath (everything that breathes has a soul, and thus, humans, plants, and animals). These communities live in total awareness of their surrounding environment, believe that they emerged from the land, and regard the natural world as nurturing life, which reflects their reciprocal relationship with the environment (Salmon 2000). Accordingly, their cultural practices and beliefs strengthen this reciprocity to the extent that they consider the environment as their extended family. Salmon (2000) further shows how environmental threats from extractive industries like logging, mining, and grazing degrade biodiversity and community well‐being by undermining the interdependent survival system between humans and nature. This kincentric worldview of the Raramuri people fosters deep forms of coexistence grounded in positive relationships with the environment.

Jolly et al. (2022) add Indigenous insights on human–wildlife coexistence from a South Indian perspective. Through a series of participatory rural appraisal workshops with Indigenous communities (Wayanad district of Kerala state in India), they find three assumed characteristics of wild animals that inform tolerance and acceptance of them: (1) rational conversing beings, (2) gods, teachers, and equals, and (3) relatives with shared origins. Jolly et al. (2022) suggest these characteristics of tolerance and acceptance as forms of deep coexistence. The ethnographic evidence from the Kattunayakans, a forest‐dwelling *Adivasi* community living in the Wayanad Wildlife Sanctuary in Kerala, shows the meaning of deep coexistence in terms of how far humans can tolerate wild animal behaviour. Kattunayakans share values towards wildlife similar to those held by the Raramuri community (Salmon 2000) and the animal agency study by Bhattacharyya and Slocombe (2017). Kattunayakans acknowledge the innate abilities of non‐human animals to understand human intentions and believe in respecting wildlife's personal space. Practices like sharing food with wildlife are considered best practices of tolerance and acceptance of wildlife, leading to deep coexistence. Importantly, the Kattunayakan and Raramuri examples are presented as illustrative examples of deep coexistence rather than representative accounts of overall HWI within a particular region/country. From a value pluralism perspective, multiple forms of coexistence and HWI may coexist within the same region or cultural contexts.

While the primary focus of this review is on IPLCs, the urban coexistence literature also provides comparative cases that illustrate coexistence principles that share values identified in IPLCs contexts, including relational responsibilities, belongingness, and deep considerations of wildlife wellbeing. The following examples are not presented as IPLC cases, but rather as comparative illustrations of coexistence that are also identified in IPLC contexts.

Deep coexistence is also found in urban ecologies. Hunold and Mazuchowski (2020) found that non‐lethal forms of urban wildlife management (i.e., preventing rodent infestations before they occur) can promote the well‐being of both humans and wildlife. They credit this management approach with strengthening human–wildlife coexistence because the non‐human animals—the wildlife—have legitimate claims on their shared spaces with humans. Aronson et al. (2017) acknowledge the significance of green infrastructure and green movements in major cities around the world, which provide pathways for hospitable environments for wildlife. Urban green spaces include parks, community gardens and cemeteries, as well as rooftop gardens and vertical gardens, meadows and woods (De Haas et al. 2021, 1). According to Aronson et al. (2017), urban green spaces play various social, cultural, and economic roles that are crucial to ensuring conservation outcomes through biodiversity management. These spaces have also been considered grounds for promoting inclusion, as they include ‘all‐natural, semi‐natural, and artificial ecological systems within and around a city’ (Aronson et al. 2017, 189), where terms like *inclusive green cities* and *just cities* have emerged (Fainstein 2013). One possibility of inclusion would be that since urban green spaces attract more non‐human animals, the animals shed their fear over time as they become desensitised, or habituated, to human presence or, as Hunold and Mazuchowski (2020) describe, their ‘misanthropy’ becomes more visible to humans.

#### 4.1.2 Thick and Thin Conception of Coexistence

In contrast to the IPLCs' behaviours with wildlife, human–animal interactions in urban spaces are considered minimal. The coexistence of urban landscapes goes beyond mere tolerance of wildlife. Michelfelder (2018) describes ethical responsibilities towards urban wildlife populations as based on a ‘parallel planes’ framework (p. 101)—the minimalist ethic of coexistence, which evokes the picture of humans and animals spending their lives scarcely encountering each other. This is identified by Hunold and Mazuchowski (2020) as a *thin conception* of coexistence. In contrast, a *thick conception of coexistence* gives a broader understanding of urban human–wildlife interactions. It requires a sense of belongingness of wildlife near humans and shared spaces. Thus, it demands that humans go beyond tolerating the presence of urban wildlife. In simple terms, drawing upon American perspectives, the coexistence of urban human–wildlife can be seen beyond tolerating wildlife since urban landscapes consider belongingness/inclusion of wildlife into their lives (Michelfelder 2018).

Consistent with the value systems attached to HWI among IPLCs, many moral norms are embedded in urban wildlife coexistence. Bekoff and Pierce (2009, 3) view these norms as moral behaviours of ‘fairness, empathy, trust and reciprocity’. Sharing green spaces, tolerating and showing a sense of belonging depend on the types of animals since they are polarised into two groups: liked and disliked (this dichotomy will be further elaborated in the proceeding section of ambivalent relationships and thinking beyond dichotomies). This variation of attitudes, meanings and behaviours ascribed to different species reflects value pluralism—different values coexisting within and across communities in shaping the relationships between IPLCs and wildlife. Exploring how far urban wildlife management reflects and accommodates human–wildlife coexistence is necessary while acknowledging wildlife as fellow urban dwellers who share legitimate claims on the spaces shared with humans (Hunold and Mazuchowski 2020).

As Hunold and Lloro (2022) demonstrated, urban wildlife must abide by the rules of humans, be tolerated, and be shown a sense of belongingness when present in shared spaces. However, some animals, like rats, are not regarded as belonging to human spaces, and lethal control is one method used to control them. As such, not all wildlife is welcome in urban spaces; reimagining urban human–wildlife coexistence without boundaries seems unrealistic. The following sections of this paper will elaborate on negative and ambivalent relationships arising from pluralistic values towards wildlife.

### 4.2 Negative Relationships Emerging Through Value Pluralism Towards Wildlife

#### 4.2.1 Community Practice of Killing Problematic Wildlife

While offering extended ways to connect with the environment, the inherent value pluralism within communities also creates conflicting relationships. Mishmi communities in Arunachal Pradesh of India, who acknowledge strong kinship ties with tigers (*Pantheria tigris*) and consider them as brothers from the same mother, offer such an example (Oommen 2021). Accordingly, if a tiger becomes dangerous, then community members will kill it (as a last resort) and consider the action as dealing with their ‘problematic brother’ (Oommen 2019, 4). Mishmi members regard this action as the most pragmatic approach, although it conflicts with India's conservation efforts and legislation (Oommen 2021).

#### 4.2.2 Lethal Control of Problematic Wildlife by Governing Institutions

There are debates about the effectiveness and cultural ethics of lethal management practices. One example of a lethal management practice enacted by a governing institution was the response to a crocodile attack in North Queensland's Bloomfield Valley in late February 2023. A saltwater crocodile ( *Crocodylus porosus* ) attacked a man and a dog on a boat ramp. The dog died, and the man was admitted to the hospital. Wildlife officers from the Department of Environment and Science euthanised the crocodile (Achenza 2023). While media coverage—primarily social and mainstream media—highlighted public empathy towards the pet dog and the man, the Indigenous community in the area, the Wujal Wujal community, reacted differently.

The Wujal Wujal community—welcome visitors and advise them about the crocodile danger that lies in the Bloomfield River. According to Evans (2023), this community emphasised that the incident could have been avoided if the man had acted more responsibly. This aligns with the values of Indian traditional societies described by Oommen (2021). Accordingly, although an animal's behaviour can be dangerous to people, that behaviour can be justified through ‘therianthropy’ (Oommen 2021, 9), where the danger to humans was due to their deliberate misbehaviour. The Wujal Wujal community members further mentioned that the death of one crocodile would change the dynamics in their entire environment and among people. Moreover, according to them, the lethal management approach to remove the danger was ineffective as it altered the stability of their environmental system. A community member expressed her feelings about the dead crocodile: ‘For us, we respect animals, our wildlife. Some of this wildlife is our totem’ (Evans 2023). Totemic species are plants, natural objects or animals gifted by a clan or a family as a spiritual emblem (Ward et al. 2023; Turner and McKenzie, n.d.). These species are of cultural significance for those gifted with the totem and considered as a protector by Aboriginal and Torres Strait Islander peoples (Ward et al. 2023). Among the many ways of showing respect to the totems is by not killing them (Frazer 2009).

Eeden et al. (2020) discuss whether removing a threat could define co‐existence by critically looking at the existing lethal and non‐lethal management strategies for dingoes ( *Canis familiaris* ) and livestock production in Australia. The ‘nil tenure’ lethal control practice, which promotes landscape‐scale poison baiting by all stakeholders, is financially and legally supported by some Australian state governments and local councils (e.g., Eeden et al. 2020, 2). Under this strategy, lethal control applies to agricultural lands and public lands, including national parks where baiting occurs in a 3‐km buffer zone of national parks to protect livestock. Moreover, the non‐lethal control called ‘cluster fencing’, where properties are fenced against pest animals covering a large area, also has some government support. However, Clark et al. (2018) show the lethal nature of this management since people tend to kill unwanted species (dingoes or other herbivores, such as kangaroos, that compete with livestock) that enter the fenced area. Eeden et al. (2020) emphasised the absence of financial support by the government to promote non‐lethal control measures like ‘livestock guardian animals or improved animal husbandry to protect livestock from dingoes in Australia’ (p. 2) and few farmers voluntarily practice those methods.

The crocodile incident in North Queensland shows the conflicting values and the different value orientations between the Indigenous communities and the management organisation but also supports scholarly findings about tolerance and acceptance behaviour by Indigenous communities (Bhattacharyya and Slocombe 2017; Jolly et al. 2022). Understanding coexistence through the lens of pluralistic values within and across different value systems enhances our understanding of the complexity and the dynamism of coexistence. So far, we have shown how pluralistic values result in positive and negative relationships while begetting and sustaining coexistence (although amidst conflicts derived due to different value orientations). In some instances, conflicting values result in mixed attitudes or behaviours towards wildlife and governing institutions of human–wildlife interactions—ambivalent relationships. Therefore, it is essential for wildlife governance to understand community values towards behaving with wildlife because these have consequences for the social sustainability of conservation efforts (Larsson et al. 2022).

### 4.3 Ambivalent Relationships Emerging Through Value Pluralism Towards Wildlife

Seeing HW‐coexistence through the lenses of value pluralism involves moving beyond dichotomous views of positive and negative relationships between humans and wildlife where positives and negatives resonate coexistence and conflicts respectively. Such a perspective provides space to acknowledge diverse and conflicting values towards wildlife among different stakeholders, including IPLCs and governing authorities. Knowing how IPLCs value a particular wildlife and existing conflicting values for the same species by governing authorities is essential in conservation planning to prevent overshadowing the underlying rationale of HWC. Although scholars have identified ambivalent relationships between humans and wildlife (Jolly and Stronza 2025; Batar 2026), limited evidence is found of how such relationships influence coexistence.

Leaving out ambivalent engagements between communities and non‐human animals creates a misleading picture of human–animal relationships (Oommen 2021). Examples in the following sections show relationships between humans and wildlife that are ambivalent but also uncertain, contradictory, and complex, and with value pluralism towards wildlife and governance of human–wildlife interactions.

The concept of kincentric ecology raises the question of how communities could consider inevitable changes, like climate change. This is addressed by Bhattacharyya and Slocombe (2017) in their study on kincentrism among the Xeni Gwet'in First Nation in British Columbia, Canada, who argue that animals and the land act as active agents that influence decision‐making processes when it comes to the co‐management of wild animals. According to these authors, considering non‐human animals as agents who make their own decisions suggests that human control of ecological relations is either non‐existent or unknowable. Since humans do not control ecological relationships, coping with uncertainty and other drivers like climate change is also beyond human control. However, Bhattacharyya and Slocombe (2017) emphasise that acknowledging uncertainty and rapid change is necessary for kincentric collaborative management.

The emphases from Bhattacharyya and Slocombe's (2017) study is consistent with Finnish and Sámi herding cultures in Finland and Sweden (Sjeogren and Matsuda 2015, 36), who historically developed various coping strategies to coexist with predators. However, the applicability of some of these strategies in modern reindeer ( *Rangifer tarandus* ) husbandry is questionable (Rasmus et al. 2020). Intensive herding, promoted by the Swedish government to make the Sámi more stationary, is one such example (Rasmus et al. 2020; Sonniksen, n.d.). Axelsson‐Linkowski et al. (2020) show that while Sámi reindeer husbandry still utilises traditional herding practices, rapid changes in the environment force them into territories where the use of traditional knowledge transmitted through generations is limited. For instance, traditional knowledge from the Sámi people highlights the importance of minimising disturbance to reindeer during winter because it affects feeding behaviour. However, with the introduction of snowmobiles for reindeer monitoring, increased tourism, and the recreational use of snowmobiles, the ‘grazing peace’ (p. 485) for reindeer is limited. Increased unpredictable weather patterns, unknown to earlier generations, also exacerbate the challenges.

### 4.4 Human–Human and Human–Wildlife Interactions and Relationship With Traditional Values

Through the concept of moral economy, Larsson et al. (2022) discuss how conflicts about wild animals between farmers and wildlife governance threaten Swedish farmers' traditional values. Essential non‐economic norms and obligations that guide the social, political, and economic relations in society are described as the moral economy (Arnold 2001). One such non‐economic obligation is reciprocity (Scott 1976). This was reflected by Larsson et al.'s study, which found that farming community members exchange various kinds of assistance during wild carnivore attacks. An example is the help that farmers receive from their neighbours and other farmers during attacks by large carnivores. They note that establishing such long‐lasting reciprocity takes time. Hence, investments in farming activities were not only about profits but also the mutual assistance (reciprocity) developed by living and historical attachments to a particular place. Scholars further relate this situation to having emotional attachments not only with a place but also with the associated values, tradition, and history during the length of the stay (Ingold 2021; Larsson et al. 2022). However, when discussing through the lenses of Larsson et al.'s (2022) and Arnold's (2001) description of moral economy, both the Swedish farmers and the traditional Indigenous communities engaging in subsistence farming share similar circumstances because they all are born into farming societies comprised of solid social relationships, moral values, and patterns of expectations about their neighbours' behaviours practised by their ancestors.

Values attributed to different wild animals differ among communities. For instance, sheep and cattle farmers in Sweden acknowledge that lynx ( *Lynx lynx* ) hunt and kill farm animals ‘as painlessly as possible’ (Larsson et al. 2022, 08) for their survival and to feed their young yet consider wolves ( *Canis lupus* ) a cruel species who hunt for pleasure rather than just for survival (Bennett et al. 2022; Larsson et al. 2022). Similarly, Finnish reindeer herders say that wolverines sometimes kill reindeer for fun rather than food (Grandma in Lapland 2013). In these contexts, wolves and wolverines have acquired a negative image compared to lynx.

Besides how humans value interactions with wildlife, Heikkinen et al. (2012)'s study based on Finnish pastoral communities shows that losses because of predators due to wildlife conflicts impact social relationships and freedom of choice. According to their study, these social relationships represent the social practices embedded in traditional herding. For instance, with the increase in wildlife depredation, herding practices change with decreased autonomy and tend to prioritise predator control and monitoring. Pursuing coexistence in situations like this requires two sets of strategies to ensure economic sustainability and the well‐being of farm animals and wildlife (Riseth et al. 2009; Rasmus et al. 2020). First is the development of novel coping strategies by herders to prevent damage. Second, adaptive management of the predator populations through more interactive strategies ensures the trust between livestock herders and policymakers (including researchers and conservationists).

As an example of value pluralism, it is worth exploring further why farmers in Sweden and Northeast Finland sympathise with the wild animals (i.e., lynx) that kill the farm animals raised for commercial gain. According to Larsson et al. (2022), although farm animals are treated as commodities, they are also raised in close relationships with farming families (e.g., especially for sheep) who ensure their death is painless. However, when wildlife like wolves hunt farm animals, the farming communities in Larsson's study express concern about the suffering of their animals. In this way, the farmers experience emotional stress and adverse financial consequences of wildlife attacks on their animals (Hultgren et al. 2014). Therefore, the already‐built relationships between farmers and farm animals also influence the values towards wildlife.

Swedish farmers and Finnish herders both encounter negative impacts from wildlife and seek mutual assistance from neighbouring farmers to overcome predation. From the Swedish farmers' point of view, they coexist with wildlife even amidst wildlife predation, but at the same time, conflict with both wildlife and wildlife governance. Finnish herders also see different wildlife with different values and have positive and negative relationships with governing institutions of human–wildlife interactions (Larsson et al. 2022). According to Swedish wildlife governance, mutual assistance to overcome wildlife predation is sometimes about hunting and harming wildlife (Larsson et al. 2022). However, acknowledging and understanding the ambivalent relationships that emerge from value pluralism highlights the coexistence of diverse values and norms in HWI.

Amidst the global environmental challenges, Hoelting (2022) emphasises the need for governance actors at local, regional, national and international scales, including community leaders, wildlife managers, and policy makers, to recognise and integrate place‐based Indigenous ways of knowing. Similarly, according to Oommen (2021), local community values should be treated seriously in decision‐making. IPLCs are not necessarily unaware of the social relationships (positive, negative and ambivalent) with wildlife, and excluding them could lead to an information deficit model. Their positive relationships are sometimes acknowledged through utilitarian mindsets, such as coexistence with wildlife in rural Japan. Sakurai (2019) provides evidence of encouraging rural locals in Japan to cook and eat wild boar ( *Sus scrofa leucomystax* ) meat to promote positive mindsets towards them. The research highlights the actions of conflict management as suggested by the locals, including capture and kill and capture and release. These examples illustrate how local values and knowledge systems can shape coexistence strategies.

## 5 Discussion: Critical Interpretation

We developed a conceptual framework to exhibit how value pluralism results in different types of relationships and interconnectedness. This framework presents and elaborates the relationships between the themes identified in the CIS. The following paragraphs explain how to understand the concepts and connections shown in Figure 3.

**FIGURE 3:**
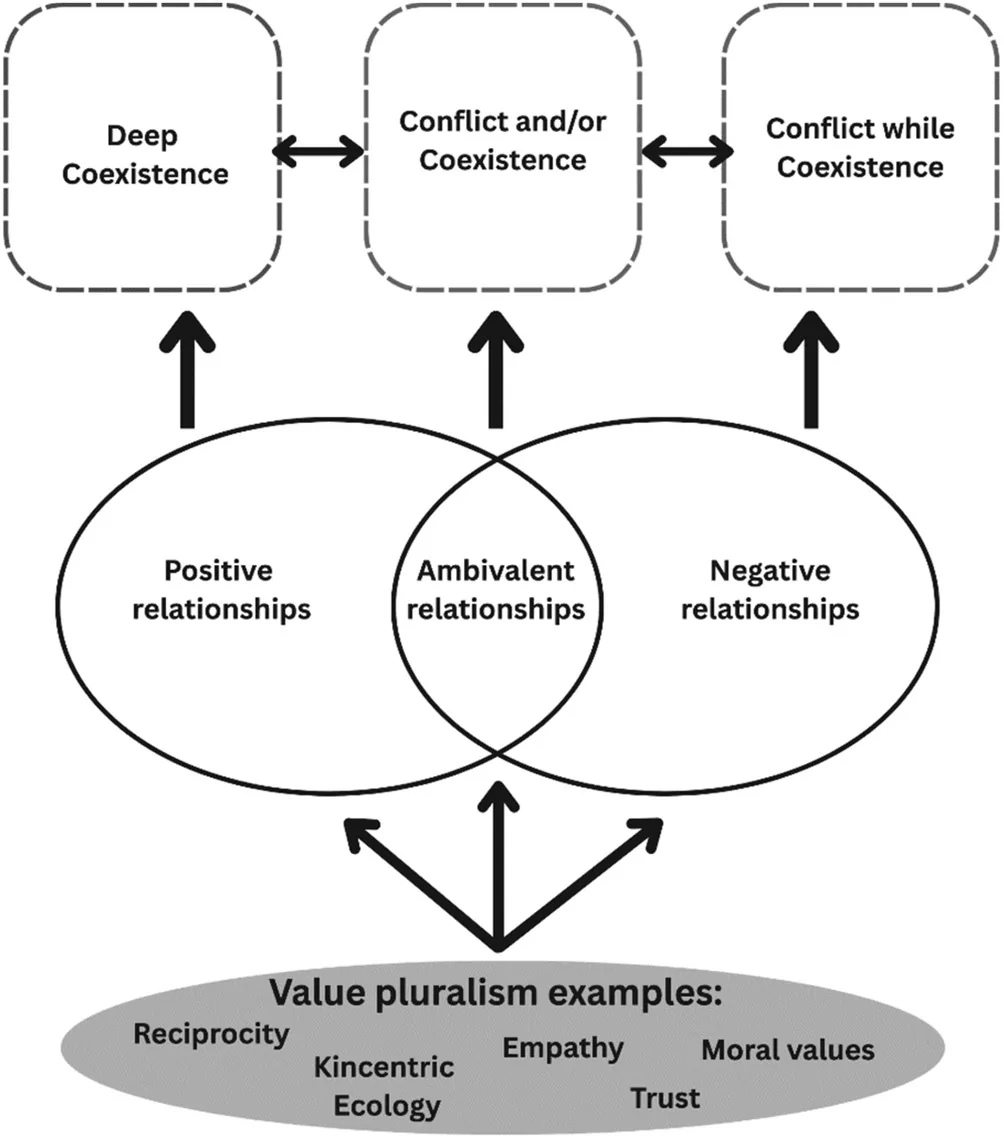
Conceptual framework for relationships derived from value pluralism. These relationships are informed by the themes identified in the CIS. Positive relationships emerge from IPLC values, including reciprocity, belongingness, and kincentric ecology, and are illustrated by themes such as deep coexistence. Meanwhile, negative relationships can also emerge from IPLC values of strong kinship ties with wildlife and are illustrated by themes such as the community practice of killing problematic wildlife and lethal control of problematic wildlife by governing institutions. Ambivalent relationships emerge from IPLC values, including trust and reciprocity, and are illustrated by themes such as human–human and human–wildlife interactions and relationship with traditional values. *Source:* authors.

The three types of relationships in human–wildlife interactions—positive, negative, and ambivalent—exist both between IPLCs and wildlife and between IPLCs and governing institutions. Although HW‐coexistence presented in the framework is in three separate squares—which are dashed—they are connected with two‐way arrows to avoid narrowing the scope and missing the gaps and connections influenced by the relationships mentioned. This also defies a simple quantitative categorisation. According to this conceptual framework, based on IPLC findings, coexistence can occur across all types of relationships between humans and wildlife. There are three types of relationships influenced by value pluralism that are intertwined and are influencing HW‐coexistence. The classification of relationship types is contingent upon the perspective from which they are evaluated. For instance, although Mishmi communities in Arunachal Pradesh of India show deep coexistence with tigers, they tend to kill tigers if they become dangerous and consider it as dealing with their ‘problematic brother’. This, from the point of view of IPLCs, is a positive relationship, but for governing authorities, this is a conflicting and negative relationship. However, viewing the relationships between humans and wildlife through value pluralism lenses, where values cannot be ranked easily, will provide a more nuanced understanding of HWI under scrutiny. Value pluralism sets the foundation in this framework and thus helps recognise multiple legitimate values held by different communities involved in HWI. This will ultimately foster a more inclusive governance approach to conservation, as the relationships and HW‐coexistence are understood beyond dichotomic perspectives.

The positive and negative relationship dichotomy, like conflict and coexistence, does not allow us to capture the nuances and complexities of interactions between humans and humans, as well as humans and wildlife. Positivity or negativity in this context is understood beyond the dichotomy of positive relationships generating coexistence and negative relationships generating conflicts. Hence, the movement between different relationships and their resulting coexistence/conflict, without confining them to single categories, represents the fluidity of the framework. Consequently, coexistence is possible in any type of relationship; for instance, while deep coexistence is found through positive relationships, negative relationships also give rise to coexistence, but in the presence of conflicts. Hence, the interactions of IPLCs with the governing institutions, as well as with wildlife, may show alignments and mismatches in their plural values that influence their coexistence outcomes. When multiple values are simultaneously invoked, ambivalent relationships emerge in which IPLCs produce uncertain, contradictory, and context‐dependent responses that cannot be understood as either positive or negative. We therefore view coexistence and conflicts as potentially overlapping and mutually fundamental aspects of HWI; consequently, they are often intertwined rather than mutually exclusive.

Understanding complexities and uncertainties within HWI, such as those mentioned among the Finnish and Sami herding cultures and Swedish farmers, requires more than simply juxtaposing positive and negative relationships. The mixed, complex and contradictory nature of ambivalent relationships gives rise to both conflicts and coexistence, but their (conflicts and coexistence) prevalence and sequence of occurrence are yet to be explored. Beyond just acknowledging the existence of ambivalent relationships and their influence on coexistence, discourse about them in conservation science is necessary to prevent them from remaining abstract concepts.

Value pluralism underlies all these engagements. Yet these ambivalent relationships and value pluralism have not been adequately recognised in HW‐coexistence literature. In this framework, value pluralism can be understood differently based on the context. For instance, the concept of kincentric ecology does not represent a singular value but rather a set of values, and these pluralistic values are found across all types of relationships and interactions, making their presence and understanding more dynamic and complex. Recognition of plural and often conflicting values as the basis of this synthesis enabled exploring their influence without constraining HW‐coexistence to tolerance and acceptance, as is widely discussed in coexistence literature. The IPBES value assessment also highlights that the relationships between humans and nature are shaped by diverse and often overlapping values, worldviews, and knowledge systems (Pascual et al. 2023). Hence, understanding human–wildlife coexistence without acknowledging value pluralism is inadequate for understanding IPLCs' interactions with wildlife.

IPLC knowledge and values towards the environment are widely acknowledged in conservation discourse, yet literature suggests that opportunities remain to strengthen their incorporation into policy and management decisions (Fletcher and Toncheva 2021). Accordingly, dominant conservation approaches are still informed by Cartesian binaries drawn between humans and nature (or else a decision between a landscape as worthy or unworthy of protection) and mask the interconnectedness between nature and the needs of present and future IPLCs (e.g., cultural, spiritual, economic) (Wiens 2007; Strang 2016). Many authors have acknowledged that the future of protected areas is dependent on the interconnectedness with IPLCs and their occupancy and use (e.g., Maezumi et al. 2018; Fernández‐Llamazares et al. 2020; Fletcher et al. 2021; Konig et al. 2021).

## 6 Conclusion

The results revealed that value pluralism towards wildlife by IPLCs enhances the dynamism and complexity of coexistence. The study has shown how value pluralism within and across different value systems gives rise to coexistence. Value pluralism exists across all socio‐ecological contexts and various community‐based groups when coexisting with wildlife. Acknowledging value pluralism, the different relationships arising from it, and the associated dynamics and complexities of coexistence must be respected and accommodated to ensure effective governance of human–wildlife interactions and achieve coexistence benefits. The longer it takes to act, the more likely it is that traditional and local knowledge will be lost, and conflicts will increase.

The conceptual framework we developed, grounded in value pluralism, portrays the fluid and overlapping nature of positive, negative, and ambivalent relationships between IPLCs and wildlife. By recognising how different values exist simultaneously, this framework supports a more nuanced and context‐sensitive understanding of coexistence between IPLCs and wildlife. Further, this framework could serve as a conceptual tool in conservation decision‐making, as it reflects the value pluralism of IPLCs beyond the reductive binaries of conflicts and coexistence of humans and wildlife. Although effective implementation depends on broad governance conditions, including institutional capacity, political will, financial resources, and sustained stakeholder engagement, which vary across contexts and scales, culturally grounded and socially legitimate conservation models are essential (Fisk et al. 2024). Hence, the suggested framework of this study holds significant potential to inform conservation policies by enhancing the understanding of the complexities and dynamics of coexistence. It supports the implementation of inclusive and adaptable conservation strategies by acknowledging the inherent significance of value pluralism within and across IPLCs interacting with wildlife.

## Author Contributions

**Anuradhi Dulangi Jayasinghe:** conceptualization (lead), data curation (lead), formal analysis (lead), methodology (lead), writing – original draft (lead). **Georgette Leah Burns:** conceptualization (supporting), methodology (supporting), supervision (lead), writing – review and editing (lead). **Duan Biggs:** supervision (supporting), writing – review and editing (supporting).

## Funding

This work was supported by Griffith University.

## Conflicts of Interest

The authors declare no conflicts of interest.

## Data Availability

The data that supports the findings of this study is included within the text (Table 1).
